# Role of Astrocytes in Parkinson’s Disease Associated with Genetic Mutations and Neurotoxicants

**DOI:** 10.3390/cells12040622

**Published:** 2023-02-15

**Authors:** Sanghoon Kim, Edward Pajarillo, Ivan Nyarko-Danquah, Michael Aschner, Eunsook Lee

**Affiliations:** 1 Department of Pharmaceutical Science, College of Pharmacy and Pharmaceutical Sciences, Florida A&M University, Tallahassee, FL 32307, USA; 2 Department of Molecular Pharmacology, Albert Einstein College of Medicine, Bronx, New York, NY 10461, USA

**Keywords:** astrocytes, Parkinson’s disease, α-synuclein, DJ-1, parkin, LRRK2, MPTP, paraquat, rotenone, 6-hydroxydopamine

## Abstract

Parkinson’s disease (PD) is a neurodegenerative disorder characterized by the loss of dopaminergic neurons and the aggregation of Lewy bodies in the basal ganglia, resulting in movement impairment referred to as parkinsonism. However, the etiology of PD is not well known, with genetic factors accounting only for 10–15% of all PD cases. The pathogenetic mechanism of PD is not completely understood, although several mechanisms, such as oxidative stress and inflammation, have been suggested. Understanding the mechanisms of PD pathogenesis is critical for developing highly efficacious therapeutics. In the PD brain, dopaminergic neurons degenerate mainly in the basal ganglia, but recently emerging evidence has shown that astrocytes also significantly contribute to dopaminergic neuronal death. In this review, we discuss the role of astrocytes in PD pathogenesis due to mutations in α-synuclein (PARK1), DJ-1 (PARK7), parkin (PARK2), leucine-rich repeat kinase 2 (LRRK2, PARK8), and PTEN-induced kinase 1 (PINK1, PARK6). We also discuss PD experimental models using neurotoxins, such as paraquat, rotenone, 6-hydroxydopamine, and MPTP/MPP+. A more precise and comprehensive understanding of astrocytes’ modulatory roles in dopaminergic neurodegeneration in PD will help develop novel strategies for effective PD therapeutics.

## 1. Introduction

Parkinson’s disease (PD) is a neurodegenerative disease characterized by the loss of dopaminergic neurons in the nigrostriatal pathway and the formation of α-synuclein aggregates (Lewy bodies) in neurons. At the clinical level, the disease is characterized by bradykinesia, gait impairment, tremor, and loss of involuntary movement [1]. PD is the second most prevalent neurodegenerative disease after Alzheimer’s disease (AD), affecting over a million Americans and causing a total economic burden in the U.S. of $51.9 billion in 2017 [2]. The etiology of PD is not well understood, but several factors appear to play a role, including gene dysfunctions and mutations. It has been shown that mutations in genes, such as leucine-rich repeat kinase 2 (LRRK2), α-synuclein, PTEN-induced kinase 1 (PINK1), and parkin, are linked to familial PD cases, but these account for only 10–15% of total PD cases (Table 1) [3]. Several cases of idiopathic PD have implicated that environmental factors, such as the pesticide rotenone and herbicide paraquat, contribute to PD development [4].

Numerous studies have investigated the molecular and cellular mechanisms underlying PD pathogenesis. Oxidative stress, inflammation, mitochondrial impairment, and dysregulation of autophagy and mitophagy have been reported in both in vitro and in vivo PD models [18,19,20,21,22]. Although dopaminergic neurodegeneration in the basal ganglia is the main feature of PD, other neural cell types, including astrocytes, have been shown to play a role in PD development [23].

Astrocytes were first described in the 19th century by Rudolf Virchow [24] and initially thought to function only in the maintenance and structural support for neurons, but recent studies have shown that astrocytes play a critical role in neurotransmitter homeostasis and synapse formation during development, potassium buffering, and maintenance of pH and the vascular lumen, just to name a few of their functions [25]. Accordingly, it is not surprising that they are implicated in multiple neurological disorders, including PD pathogenesis and progression [26,27]. Hence, studying the functional role of astrocytes is important for understanding the full spectrum of PD pathogenesis and for the development of novel therapeutic strategies to treat PD. In this review, we discuss the role of astrocytes in both genetic and neurotoxins-induced PD pathology, as well as potential neuroprotective strategies targeting astrocytes in PD.

## 2. Role of Astrocytes in PD-Related Gene Mutations-Induced Pathology

### 2.1. DJ-1

DJ-1 is encoded by the PARK7 gene, and its mutations are one of the causes of autosomal-recessive PD [28,29,30]. DJ-1 is expressed abundantly in astrocytes compared to neurons, and it is further upregulated in reactive astrocytes in chronic neurodegenerative disorders, including PD [31,32]. DJ-1 is associated with lipid rafts in astrocytic plasma membranes [11]. Lipid rafts are involved in many cellular signaling processes, such as membrane receptor trafficking, endocytosis, and signal transduction [33]. Deficient DJ-1 activity due to PARK7 gene mutation increases the degradation of the lipid raft proteins and, thus, disrupts lipid raft assembly [12]. DJ-1-deficient astrocytes display impaired glutamate uptake [12] since astrocytic glutamate transporter excitatory amino acid transporter 2 (EAAT2) protein is assembled in lipid rafts [34]. In turn, EAAT2 dysfunction leads to excess levels of synaptic glutamate and excitotoxic neuronal injury, which is related to a variety of neurodegenerative diseases [35], including PD. Lipid rafts are also involved in astrocytic immune responses to inflammatory stimulation, and thus, the disruption of lipid raft assembly in DJ-1 deficient astrocytes could increase inflammatory cytokine production in response to lipopolysaccharide (LPS) [11,36] and increase neuronal toxicity. The role of astrocytic DJ-1 in PD is supported by experimental PD models. Studies have shown that neurotoxic effects induced by PD toxicant models, such as rotenone and 6-hydroxydopamine (6-OHDA), caused greater cytotoxicity to neurons when co-cultured with DJ-1 deficient-astrocytes than those with wild-type (WT) astrocytes [37,38]. Moreover, overexpression of DJ-1 in astrocytes affords neuroprotection against rotenone toxicity [37].

In addition to its role in lipid raft signaling and glutamate uptake in the plasma membrane, astrocytic DJ-1 is critically involved in maintaining normal mitochondrial function as well as slowing PD progression by modulating mitochondrial motility and fission [39]. Studies have shown that mitochondrial dysfunction in astrocytes is closely associated with aberrant glutamate metabolism and excitotoxicity [40,41,42], suggesting that DJ-1 function in mitochondria may be inter-regulated with glutamate transporter in astrocytes. Moreover, the effect of DJ-1 dysfunction on mitochondria caused intracellular oxidative stress, activating the antioxidant transcription factor nuclear factor erythroid 2-related factor (Nrf2) and Nrf2’s target genes [41,43,44]. Additionally, DJ-1 deficiency induces inflammatory activation in astrocytes [45]. It also impairs monocyte infiltration into the damaged brain owing to a decrease in the astrocyte-specific chemokine, chemokine (C-C motif) ligand 2 (CCL2) levels in mice [46], resulting in further inflammation and the delay of neuronal repair. 

### 2.2. α-Synuclein

The α-Synuclein is encoded by the SNCA gene, and the aggregates of this protein caused by its mutations are a pathological hallmark of PD. The role of α-synuclein aggregates in neurons has been extensively studied [47], but their spreading to adjacent astrocytes and, thus, consequential toxic effects via astrocytes remain largely unexplored. The α-synuclein is predominantly expressed in presynaptic nerve terminals, playing an important role as a molecular chaperone in providing an adequate supply of synaptic vesicles in presynaptic terminals, assisting in the folding and refolding of synaptic SNARE proteins, the release of neurotransmitters, such as dopamine, and synaptic integrity [48], to name a few of its functions.

Astrocytes are essential for fatty acid metabolism in the brain [49]. Although the abundance of α-synuclein in astrocytes is low in comparison to neurons [50], it has been shown to play a role in fatty acid metabolism, which is important in energy homeostasis, membrane maintenance, and cell signaling. These suggest that α-synuclein may regulate fatty acid metabolism in astrocytes. Studies have shown that α-synuclein deficiency decreased the rate of arachidonic acid and palmitic acid metabolism in astrocytes, suggesting that α-synuclein and lipid metabolism in astrocytes may be implicated in PD pathology [51,52,53].

That astrocytes regulate exogenous α-synuclein is supported by the finding that α-synuclein-containing inclusions were found in astrocytes of postmortem PD brains [54]. Astrocytes take up α-synuclein secreted from neurons [55] via a toll-like receptor 4 (TLR4)-independent endocytosis pathway [5] and localize this abnormal protein into the lysosomes [56], suggesting that astrocytes have a role in its removal and degradation. Moreover, high levels of extracellular α-synuclein have been shown to induce a TLR4-dependent inflammatory response in primary astrocyte cultures [5], indicating a possible role of astrocytes in α-synuclein-induced inflammatory PD pathology. This accumulated astrocytic α-synuclein may dysregulate critical astrocytic functions, such as glutamate uptake, by decreasing the expression of the astrocytic glutamate transporters, glutamate-aspartate transporter (GLAST, EAAT1 in human), and glutamate transporter 1 (GLT-1, EAAT2 in human), as well as blood–brain barrier (BBB) integrity by disrupting the localization of the water channel Aquaporin-4 (AQP4). Mice, in which mutant α-synuclein was specifically overexpressed in astrocytes, developed astrogliosis prior to the onset of symptoms with dopaminergic neurodegeneration in substantia nigra pars compacta [6].

Since the α-synuclein aggregates are injurious pathologic factors in PD progression, several therapeutic interventions using astrocytes have been developed to effectively eliminate them. Studies on neuron-astrocyte co-cultures have shown that astrocytes from the midbrain inhibited neuronal α-synuclein aggregation and its transmission to other cells in a paracrine mode, resulting in attenuation of the α-synuclein-induced toxicities. Similar observations were made in an in vivo PD mouse model in which transplantation of healthy midbrain astrocytes into the midbrain of the α-synuclein expressing PD mice attenuated α-synuclein pathology and dopaminergic toxicity [57].

The astrocytic autophagy-lysosomal function could also be targeted to eliminate α-synuclein since this astrocytic function is important in the degradation of α-synuclein taken up by astrocytes. Moreover, the efficiency of this pathway is dependent on the astrocytic function of ATP13A2 [58], mutations of which are linked to autosomal recessive PD pathology, supporting the role of ATP13A2 in astrocytic lysosomal degradation of α-synuclein aggregates. The functional role of ATP13A2 in astrocytes is discussed in the next section.

### 2.3. ATP13A2

ATP13A2, encoded by PARK9, is a transmembrane lysosomal ATPase. It is highly expressed in the brain, particularly in the substantia nigra pars compacta [59], consistent with involvement in the pathophysiology of PD. Mutations in the PARK9 gene impair lysosomal functions and are linked to autosomal recessive early-onset parkinsonism [59,60]. The ATP13A2 mutations lead to abnormal α-synuclein aggregation and oxidative stress in mitochondria [61].

ATP13A2 regulates the astrocytic uptake and degradation of α-synuclein released by neurons. Loss-of-function mutations in ATP13A2 impair α-synuclein clearance by astrocytes and lead to the activation of nod-like receptor protein 3 (NLRP3) inflammasomes in astrocytes [61]. Given the protective role of astrocytes in decreasing the neuronal accumulation of α-synuclein, these protective functions are decreased in astrocytes carrying ATP13A2 mutations, resulting in increased accumulation and propagation of α-synuclein, as was demonstrated when induced pluripotent stem cell (iPSC)-derived dopaminergic neurons were cocultured with astrocytes from either healthy subjects, or patients carrying mutations in lysosomal *ATP13A2* [58].

### 2.4. LRRK2

LRRK2, encoded by the PARK8 gene, is a large 286 kDa protein with dual kinase and GTPase activity [62,63]. It regulates multiple functions, such as trafficking, stress response, autophagy, and inflammation [64,65,66]. Mutations in LRRK2, such as G2019S, R1441C/G/H, and I2020T, are the most common genetic cause of late-onset PD [67]. They are autosomal dominant [62,68] and induce hyper LRRK2 kinase activity. Activation of the LRRK2 kinase activity, even independent of its mutations, is also known to contribute to the pathogenesis of idiopathic PD [69], implicating LRRK2 in both familial and sporadic PD cases. LRRK2 is expressed in neurons, astrocytes, and microglia in the human brain [70], with astrocytic LRRK2 being localized primarily in lysosomes to regulate lysosome size, number, and function, as well as the autophagy-lysosomal pathway for the degradation and recycling of cellular components [14,15,66]. In physiological conditions, autophagy proceeds with the lipidation of the LC3-I protein to the LC3-II protein, which is then transported to the membrane of autophagic vesicles for the normal autophagy process. However, increasing LRRK2 kinase activity decreases the lipidation of LC3-I in primary mouse astrocytes [14], leading to dysregulation of autophagosome formation. 

G2019S, R1441C, and Y1699C LRRK2 mutations induce enlarged lysosomes in astrocytes, impair lysosomal degradation of long-lived and damaged proteins, and reduce lysosomal pH due to increased LRRK2 kinase activity [15]. 

Interestingly, while “normal” astrocytes express low levels of α-synuclein, cultured iPSC-derived astrocytes carrying the PD LRRK2 G2019S mutation exhibit progressive endogenous α-synuclein accumulation, dysfunctional chaperone-mediated autophagy, and impaired macroautophagy, resulting in the decreased astrocytic ability to clear neuronal-derived extracellular α-synuclein aggregates when co-cultured with WT dopaminergic neurons [71]. Moreover, astrocytes with the LRRK2 G2019S mutation transferred α-synuclein to neighboring neurons, leading to α-synuclein accumulation and neurodegeneration, suggesting that LRRK2 mutated astrocytes can mediate toxicity in the course of PD pathogenesis [71].

Moreover, astrocytes with LRRK2 G2019S mutation show dysregulation of the actin-binding protein annexin A2, which is also a novel player in α-synuclein internalization by astrocytes, resulting in impairment of the astrocytic endo-lysosomal pathway to clear extracellular fibrillar α-synuclein [72].

### 2.5. PINK1 and Parkin

#### 2.5.1. PINK1

PINK1, encoded by the PARK6 gene, is a protein involved in mitophagy, a process that selectively degrades damaged mitochondria following mitochondrial stress [73]. PINK1 loss-of-function mutations are linked to recessive PD cases [74]. The role of astrocytic PINK1 is underscored by the findings that activation of PINK1 by mitochondrial injury occurs predominantly in astrocytes among all neural cell types in the CNS [68], while it is almost absent in neurons [75]. This suggests that astrocytes represent a key neural cell type affected by PINK1 deficiency in familial PD cases [68]. Conditioned media from LPS/IFN-γ-treated PINK1-deficient astroglia induced a higher apoptotic rate in neurons compared to media from treated WT astroglia, suggesting that astrocytic PINK1 is important to protecting neurons against apoptotic neuronal injury [76]. Additionally, PINK1 expression in the brain is increased during embryonic development and has an important role in the development of astrocytes [77] and the cyto-protection against oxidative stress-induced apoptosis [76].

#### 2.5.2. Parkin

Parkin, encoded by PARK2, is a cytoplasmic E3 ubiquitin ligase that plays a crucial role in mitophagy and clearance of reactive oxygen species (ROS) [78]. Parkin mutations are well known to be linked to the autosomal recessive PD. Constitutive Parkin expression is high in neurons as compared with astrocytes, but unfolded protein stress elicits a selective increase in astrocytic Parkin expression and a change in its distribution, while neuronal Parkin remains largely unchanged. Moreover, in stress conditions that cause unfolded protein production, protein levels of Parkin increase in astrocytes, but not in neurons, leading to neuroprotective effects and thus decreasing neuronal injury [79], suggesting the astrocytic Parkin’s role in neuroprotection against stress condition, and pathogenic role of astrocytes in PD carrying a Parkin mutation. In mouse primary midbrain astrocyte cultures, Parkin deficiency led to the presence of fewer astrocytes by reducing their proliferation and increasing proapoptotic protein expression compared to WT controls. In addition, astrocytic Parkin deficiency caused structural mitochondrial defects [8], and exacerbated α-synuclein-induced impairment of mitochondrial respiration [80].

#### 2.5.3. PINK1/Parkin Mitophagy

PINK1 is well known to play a critical role in mitophagy, in collaboration with Parkin in the PINK1-Parkin mitophagy pathway, which removes damaged mitochondria [81]. PINK1 activity induces Parkin translocation from the cytosol into mitochondria to bind depolarized mitochondria [81]. Transcellular mitophagy (transmitophagy), which is a mitochondrial transfer from neurons to astrocytes in the context of neuronal injury, occurs primarily in neighboring astrocytes to degrade axonal mitochondria [82]. Trans-neuronal mitophagy occurs in in vivo rat and mouse PD models [83], and astrocytes are the primary cell type responsible for the clearance of damaged mitochondria from neurons. These findings suggest that boosting PINK1/Parkin-mediated mitophagy in striatal astrocytes may help with the removal of damaged mitochondria in dopaminergic neurons.

Moreover, optimal mitophagy function can eliminate dysfunctional mitochondria to maintain mitochondrial homeostasis and protect against neuroinflammatory activation induced by ROS [84], indicating that impaired mitophagy induced by mutations in PINK1 and Parkin can provoke inflammatory activation in astrocytes [40]. There is a possible correlation between mitochondria perturbations and astrocyte inflammatory activation. Impaired mitophagy from PINK1 or Parkin mutations in neurons and glia may promote mitochondrial fragmentation and enhance inflammatory responses in astrocytes, leading to the potentiation of inflammatory activation and dysfunction of neighboring cells. Thus, the removal of damaged mitochondria from astrocytes may be broadly neuroprotective and beneficial against undesired chronic neuroinflammation in PD [85].

Impaired PINK1/Parkin-mediated mitophagy can trigger NLRP3 and other inflammatory pathways [85] by the mitochondria-specific lipid cardiolipin. Mitochondrial depolarization results in the translocation of cardiolipin from the inner mitochondrial membrane to the outer mitochondrial membrane, where it associates with NLRP3 [86]. An attractive hypothesis is that in PINK1 and Parkin mutant astrocytes, elevated levels of mitochondrial ROS and cardiolipin result in the sustained activity of the NLRP3 inflammasome. Notably, there are reports indicating that in vitro PINK1/Parkin mitophagy can be inhibited upon inflammasome activation since Parkin can be cleaved by caspase-1 and caspase-8 [87,88]. Collectively, these studies demonstrate the crucial role that mitochondria play in the activation of NLRP3 and underline the PINK1/Parkin pathway of mitophagy as a key mechanism limiting excessive inflammation and preserving CNS homeostasis.

Impaired mitophagy in Parkin- and PINK1-deficient astrocytes might further lead to a decrease in astrocyte proliferation and decreased GFAP-positive astrocytes in the substantia nigra, potentially contributing to the development of PD due to the delay of astrocyte-mediated repair of the brain microenvironment [10,77].

### 2.6. Glucocerebrosidase

Glucocerebrosidase (GCase), encoded by the *GBA1* gene, is a lysosomal enzyme that is involved in glycolipid metabolism. Heterozygous carriers of specific mutations in this gene are at an elevated risk of developing PD [89]. Human and murine astrocytes express high levels of this protein, suggesting its critical role in this cell type [90].

GCase is involved in lysosomal function, playing a role in autophagy. The iPSC-derived astrocytes carrying mutations in the *GBA1* gene exhibited astrogliosis with impaired lysosomal cathepsin activity, resulting in the accumulation of α-synuclein aggregates [91]. These results are supported by other studies reporting that GBA-deficient primary astrocytes showed defective autophagic and proteasomal machinery [92]. GBA-deficient mice also showed impairment of their autophagic pathway, as evidenced by reductions in both LC3-I and LC3-II fragmentation [93]. These studies demonstrate that astrocytic GCase plays a role in autophagy, and its functional deficiency due to mutations can result in the accumulation of toxic proteins in astrocytes, thus indirectly causing neurotoxicity. On the other hand, mutation of GBA1 in mouse primary astrocytes did not affect the degradation of α-synuclein, despite resulting in a decrease in lysosomal protease activity [94]. This indicates that the effects of astrocytic GBA1 deficiency could differ depending on the experimental setting, although the astrocytic role of GCase function in autophagy is consistent.

GCase-deficient astrocytes due to either GBA1 mutations or deletion are closely associated with astrogliosis and neurodegeneration in a mouse model [95]. Moreover, GBA1-deleted mice exhibited astroglial activation within the nigrostriatal pathways, accompanied by the accumulation of α-synuclein aggregates [96]. Abnormal α-synuclein accumulation was also detected clinically in post-mortem brain tissue from patients carrying a mutant GBA1 [89,97]. These findings suggest that GBA1-mutated astrocytes play a pathogenic role in astrocytic inflammatory responses, possibly contributing to neuronal injury and an increased risk of PD. Moreover, GBA1 mutations in primary astrocytes decreased immune responses by reducing several pro-inflammatory cytokines when exposed to LPS [94]. Although the role of *GBA1 mutation*-induced decrease in the production of cytokines in PD is not well understood at present, it may be similar to the decreased cytokine responsiveness observed in aging, which is the greatest risk for PD. GBA1 mutation-induced immune response dysregulation in astrocytes was normalized by inhibiting LRRK2 kinase activity, indicating functional intracellular crosstalk between GCase and LRRK2 in astrocytes [94].

GCase also plays a role in maintaining mitochondrial functions, such as mitophagy in astrocytes [92]. GBA1-deficient mouse primary astrocytes exhibited mitochondrial dysfunction characterized by reduced ATP generation, decreased membrane potential, and mitochondrial fragmentation, possibly due to impaired mitophagy caused by lysosomal defect [92,93].

### 2.7. Prospectives

Astrocyte reprogramming studies have shown some promising strategies by which astrocytes in the CNS can be transformed into functional neurons. These strategies are applicable to many gene mutations related to PD, including those in the α-synuclein gene (reviewed in [98]). Reprogramming methods, targeting specific transcription factors and mRNAs by modulating astrocytic microRNA (miRNA) mechanisms in the mouse brain, have been able to reprogram astrocytes into functional dopaminergic neurons (reviewed in [99]).

## 3. The Dysregulations of Astrocytic Cellular and Molecular Mechanisms in Experimental PD Models

The protective role of astrocytes for dopaminergic neurons in PD is well established by several studies. For example, 6-OHDA-induced dopaminergic neurodegeneration was exacerbated in the absence of astrocytic function, which was induced by chronic infusion of fluorocitrate into the substantia nigra of the rat brain [100]. Moreover, without astrocytic support, the substantia nigra displayed a higher microglial activation and reduced survival capability of the dopaminergic system in the 6-OHDA rat model [100]. This indicates that dopaminergic neurons in the substantia nigra pars compacta are more vulnerable to PD toxicants in the absence of astrocytic support. Extensive studies have been conducted to determine the astrocytic factors that modulate PD development. These include oxidative stress, inflammation, synaptic dysfunction, endoplasmic reticulum (ER) stress, trophic factor reduction, and apoptosis in astrocytes. PD neurotoxicants, such as paraquat, 1-methyl-4-phenyl-1,2,3,6-tetrahydropyridine (MPTP)/1-methyl-4-phenylpyridinium (MPP+), 6-OHDA and rotenone, have been extensively studied in astrocytes, in addition to neurons, to understand the role of astrocytes in the pathogenesis of idiopathic PD [37,38,101,102]. The following sections will focus on how these neurotoxins contribute to PD pathogenesis by affecting astrocytic cellular and molecular mechanisms.

### 3.1. The Role of Astrocytic Oxidative Stress in PD Development

Oxidative stress is a well-known important contributor to the progression of neurodegenerative disorders [103]. ROS are mainly produced in the mitochondria via oxidative phosphorylation and electron transfer reactions. Abnormal ROS production causes excessively abnormal peroxidation of lipids and proteins, resulting in DNA oxidation and strand breakage, leading to cellular injury. It has been shown that PD patients’ blood and cerebrospinal fluid (CSF) have higher levels of oxidative stress compared to healthy subjects [103]. Causal factors of PD-related oxidative stress may include (1) impairment of mitochondrial function and degradation through genetic mutations and various toxicities and (2) dysregulation of antioxidant defense capabilities in the brain.

ROS produced by astrocytes are implicated in oxidative stress in various PD models, including primary neurons and astrocytes [103,104,105,106], leading to neuronal injury and PD-like neurological deficits in animals. Most PD-like neurotoxicants, such as rotenone, MPTP/MPP+, and 6-OHDA, trigger ROS production in astrocytes [13,38,107,108,109,110,111], and the ROS production is likely derived from the impaired mitochondrial function and dysregulated astrocytic antioxidant defense mechanisms.

Dysregulation of the astrocytic antioxidant system is well known to augment neuronal stress responses in primary astrocyte and mouse PD models [112]. Studies have shown that the expression of the antioxidant transcription factor Nrf2 and its target genes in astrocytes is reduced in an MPTP-induced PD mouse model [113], while overexpression of astrocytic Nrf2 protects neurons against MPTP-induced dopaminergic neurotoxicity in mice [113]. Activation of astrocytic Nrf2 also attenuated 6-OHDA-induced neurotoxicity in rats and Drosophila [114]. Moreover, the mutation of DJ-1, which causes an autosomal recessive PD, is closely associated with Nrf2 dysfunction. This is supported by the findings that DJ-1-deficient or -mutated astrocytes caused Nrf2 instability, reducing its transcriptional activities [115], and abolished astrocyte-mediated neuroprotection against PD [13,38]. However, DJ-1 overexpression in astrocytes protected dopaminergic neurons against rotenone-induced neurotoxicity in the substantia nigra of rat brain [107], suggesting that astrocytic oxidative stress plays a critical role in PD, at least in part by dysregulating Nrf2 and DJ-1. In addition, the co-culturing of primary rat astrocytes with neurons increased neuronal glutathione (GSH), preventing paraquat-induced damage to neurons [112]. Moreover, 6-OHDA-induced neuronal death was exacerbated in mesencephalic cultures pre-cultured with GSH-depleted astrocytes, but not complex I-inhibited astrocytes, compared to co-culturing with normal astrocytes. MPTP-induced neurotoxicity in mice was exacerbated by a deficiency in astrocytic SMP30, an essential component of the antioxidant vitamin C production [116]. These findings suggest that excessive astrocytic ROS may increase neuron vulnerability [117] and that enhancing astrocyte antioxidant defense systems could be an effective strategy for reducing oxidative stress in PD.

### 3.2. The Role of Astrocytic Inflammation in PD Development

Inflammation was first observed in the substantia nigra pars compacta of PD patients in the 1980s [118]. Later, more studies demonstrated that post-mortem brains, CSF, and serum of PD patients showed higher levels of proinflammatory cytokines, such as interleukin (IL)-1β, tumor necrosis factor (TNF)-α, IL-2, and IL-6, compared to those in healthy controls [119]. Although microglia are well-known inflammatory cytokine producers, astrocytes also release cytokines and inflammatory mediators under toxic conditions, leading to inflammation and cell death in dopaminergic neurons [120,121,122]. Numerous studies have reported the involvement of astrocytes in the MPTP neurotoxicity model, as MPTP triggered proinflammatory astrocytic activation and subsequent neurotoxicity [121]. This was supported by the findings that the inhibition of astrocytic activation in mice with iptakalim attenuated MPP^+^-induced astrocytic inflammation as well as dopaminergic toxicity in the substantia nigra [121]. By using ALDH1L1 bacTRAP mice to identify genes being expressed in astrocytes, MPTP was observed to induce the production of the cytokines IL-1β, TNF-α, and CCL4 [123]. In addition to producing cytokines, evidence has shown that inflammatory reactive astrocytes amplify microglial activation under MPTP exposure [120], suggesting that astrocyte-microglia crosstalk contributes to neuroinflammation. Arundic acid, which exclusively suppresses astrocyte activation [124], attenuated MPTP-induced reactive astrocytes and dopaminergic neurotoxicity in mice [101,125]. Another PD toxin, 6-OHDA, was also shown to increase inflammatory astrocyte reactivity and expression of proinflammatory molecules, including inducible nitric oxide synthase (iNOS), nitric oxide (NO), cyclooxygenase 2 (COX-2), prostaglandin E2, and TNF-α in primary astrocytes [126]. A role for iNOS is indicated as its inhibition attenuated 6-OHDA-induced mitochondrial impairment and apoptosis in C6 astrocytes [127]. These findings indicate that astrocytes play a substantial role in mediating neurodegeneration via inflammation [102].

Studies have shown that astrocytic aquaporins and secretogranins are involved in the production of bioactive compounds from the cells in PD models. Paraquat at high exposure concentrations increased the expression of both secretogranin II and IL-6 and their colocalization in U118 astrocytes [47], while inhibition of secretogranin II decreased IL-6 protein levels in astrocytes, suggesting that astrocytic secretogranin could influence inflammation by regulating IL-6 production and release. Moreover, AQP4, a water-selective membrane channel regulating BBB permeability and immune response, is involved in MPTP-induced production of IL-1β and TNF-α in primary astrocyte cultures [120]. This is supported by the results that deletion of AQP4 exacerbated inflammatory astrocyte reactivity and levels of these cytokines, along with enhanced NF-κB activation in MPP^+^-treated astrocytes [120] and amplified microglial activation in astrocyte-microglia co-cultures. These data indicate that astrocytic aquaporins play a critical role in the attenuation of astrocytic inflammation and microglial activation, serving as a potential target for treating MPTP neurotoxicity.

### 3.3. Dysregulation of Astrocytic Degradation of Damaged Proteins in PD Development

Eliminating protein aggregates, such as the Lewy bodies that can be formed by misfolded α-synuclein, is essential for the cell’s health and physiological functions [128]. Several biological processes degrade damaged proteins and toxic substances, including endo-lysosomes, autophagy, and the ubiquitin-proteasomal pathway [129]. Given that α-synuclein could spread from neurons to astrocytes [56,130], astrocytes play a crucial role in removing α-synuclein via the activation of lysosomes and lysosomal enzymes [131].

Accumulating evidence suggests that astrocytes play a major role in removing accumulated debris caused by the degeneration of synapses and axons of dopaminergic neurons in a PD animal model [132]. Astrocytes can take up extracellular debris and damaged components via phagocytosis and endocytosis [56,133] and degrade these materials in lysosomes [134]. PD-related mutations in LRRK2, such as G2019S, R1441C, and Y1699C, cause lysosomal defects and abnormal lysosomal morphology that reduce degradative capacity in astrocytes [15], leading to the accumulation of cellular debris and cell death in astrocytes. A similar reduction in autophagic capacity in astrocytes is caused by neurotoxicants that produce PD-like symptoms, such as rotenone and paraquat. High concentrations of paraquat initially activate autophagy in astrocytes but subsequently reduce autophagosome formation and autophagic flux, leading to the accumulation of toxic substances in astrocytes [135]. These toxic substances can spread to neighboring cells, including neurons, by several mechanisms, such as exosomal propagation [136], passive transport [133], direct contact, and tunneling [137].

As described in earlier sections, loss-of-function mutations in ATP13A2 (PARK9) are associated with PD (for a review, see [138]). In physiological conditions, ATP13A2 regulates the formation and secretion of intraluminal vesicles and exosomes [138]. However, ATP13A2 mutations cause abnormal α-synuclein accumulation in neurons [139,140]. Moreover, ATP13A2 mutations reduce the ability of astrocytes to eliminate α-synuclein, resulting in α-synuclein accumulation in astrocytes and interneuronal transfer of α-synuclein in iPSC-derived dopaminergic neurons, damaging neurons in the astrocyte-neuron co-culture [58].

Rotenone induces inflammatory reactive astrocytes, and phosphorylated α-synuclein aggregates in dopaminergic neurons prior to dopaminergic neuronal loss in the substantia nigra [141], indicating that activation of astrocytes and aggregation of phosphorylated α-synuclein precede neuronal loss in rotenone neurotoxicity. 

These findings indicate that targeting astrocytic degradation and clearance of protein aggregates could be an important therapeutic strategy for preventing the accumulation of toxic substances in PD.

### 3.4. The Role of Astrocytes in Inducing Excitotoxicity in PD Development

Glutamate is the main excitatory neurotransmitter in the CNS, inducing excitatory synaptic transmission, followed by its uptake into the adjacent astrocytes [142]. Studies have shown that excessive glutamate neurotransmission may be involved in PD pathogenesis, as the N-methyl-D-aspartate (NMDA) receptor antagonist, Ro 25–6981, attenuated Parkinsonian motor symptoms in the MPTP-induced PD mouse model [143].

Astrocytes maintain proper homeostasis of glutamate neurotransmission via glutamate-glutamine metabolism and glutamate uptake from the synaptic cleft via glutamate transporters, such as GLAST (EAAT1) and GLT-1 (EAAT2). Accordingly, the dysregulation of astrocytic glutamate transporters may lead to excessive glutamate accumulation in the extracellular synaptic cleft, leading to overstimulation of postsynaptic glutamate receptors and, thus, excitotoxic neuronal cell death. Although glutamate uptake cannot be measured in the brain of PD patients while alive, studies have shown that PD patients had a 48% (*p* < 0.0001) lower glutamate uptake in platelets compared to healthy controls [144], which correlated with increased severity of parkinsonian symptoms (*p* < 0.05). Although there is no report linking platelet glutamate uptake to astrocytic glutamate uptake, this finding implicates the potential dysfunction of glutamate uptake in PD.

Studies have shown that PD-related gene mutations, such as DJ-1 and LRRK2, are associated with impaired glutamate uptake in astrocytes resulting from impaired GLT-1 trafficking and localization and transcriptional repression [12,145]. The LRRK2 G2019S mutation decreased EAAT2 expression in PD human brains [145]. LRRK2 G2019S also reduced GLT-1 in the striatum of mice and primary astrocytes at the transcriptional and posttranslational levels, leading to a reduction in glutamate uptake [145]. Moreover, LRRK2 G2019S caused GLT-1 sequestration in Rab4-positive vesicles, preventing GLT-1 localization in the plasma membrane of primary astrocytes [145], leading to abnormal GLT-1 trafficking and increased degradation [12]. Nedd-4-mediated ubiquitination of GLT-1 decreased GLT-1 expression in an MPTP-PD mouse model, while its knockdown reversed the MPTP-induced decrease in GLT-1 expression and reduced both motor deficits and dopaminergic cell loss [146]. A 6-OHDA decreased striatal GLT-1 levels along with an increase in PD-like deficits [147]. Paraquat and MPTP also reduced astrocytic GLT-1 expression as well as glutamate uptake and led to dopaminergic neurotoxicity in mice [142]. In addition, an upregulation of EAAT2 increased glutamate uptake in human U251 astrocytes and afforded more protection against 6-OHDA toxicity in the neuroblastoma SH-SY5Y cell line [148].

### 3.5. Dysregulation of Astrocytic Growth Factor Synthesis in PD Development

Neurotrophic factors, such as brain-derived neurotrophic factor (BDNF), ciliary neurotrophic factor (CNTF), epidermal growth factor (EGF), acidic fibroblast growth factor (FGF), insulin-like growth factor (IGF), transforming growth factor-alpha (TGF-α), and glial cell line-derived neurotrophic factor (GDNF), have been shown to promote neuronal health and survival (for review, see [149]). Studies have shown that BDNF and nerve growth factor concentrations were decreased in the substantia nigra in PD patients [150,151], suggesting that growth factors were decreased in the PD brain.

Enhancing astrocytic levels of BDNF and GDNF ameliorated dopamine neurodegeneration induced by MPP^+^ in rat-derived primary cultures of astrocytes and neurons [152], underscoring the role of astrocytic growth factors in neuroprotection. In the 6-OHDA PD animal model, astrocytic trophic factors were decreased, which contributed to neuronal injury. This is supported by the result that 6-OHDA inhibited ATP-dependent Ca^2+^ signaling, which is critical for trophic factor production in astrocytes [153]. It is well established that astrocytes afford a protective role in PD by supplying neurotrophic factors, such as nerve growth factor and BDNF [154], and that these protective effects are decreased in advanced PD [155].

Astrocytic growth factors induce neuroprotection by multiple cellular mechanisms. Healthy astrocytes elicit protective effects against MPTP-induced inflammation via IGF-1, IGF-1 receptor, and G protein-coupled estrogen receptor (GPER) in mice [156]. Astrocytic IGF-1 has been shown to attenuate MPP^+^-induced upregulation of COX-2 and iNOS protein levels in primary astrocytes by binding to the IGF-1R and activating the GPER/PI3K/MAPK signaling pathway [156]. Astrocytic GDNF and BDNF have also been reported to protect neurons against 6-OHDA toxicities [157,158], although the efficacies of these trophic factors have not been conclusive [159,160]. The mesencephalic astrocyte-derived neurotrophic factor (MANF) [161] has been shown to offer neuroprotection in PD 6-OHDA-treated neuronal cell lines, exerting antioxidative stress, anti-inflammation, and antiapoptotic properties [122,162,163,164], as well as increasing antioxidant proteins Nrf2/HO-1 and Wnt signaling [122]. These findings imply that a reduction in astrocytic trophic factors contributes to adjacent neuronal injury, leading to exacerbation of nigrostriatal dopaminergic neurodegeneration in PD [117,153].

### 3.6. The Role of Astrocytic ER Stress in PD Development

In healthy cells, the ER regulates protein folding and trafficking, and thus, acute perturbations to ER homeostasis can alter the folding process, causing ER stress and inducing the unfolded protein response (UPR) to restore ER homeostasis and function [165]. However, chronic ER stress could lead to the accumulation of unfolded proteins, such as α-synuclein, and prolonged UPR activity can result in apoptotic cell death. PD brain tissues have shown accumulation of α-synuclein in the ER [166,167], suggesting that chronic ER stress is associated with α-synuclein misfolding and accumulation.

It has been shown that astrocytic ER stress triggered by excessive α-synuclein uptake leads to Golgi apparatus fragmentation and apoptosis in primary rat astrocytes [168]. Excessive α-synuclein impairs the normal degradation process in astrocytes, leading to higher α-synuclein accumulation in the trans-Golgi network and ER stress [137]. Moreover, LRRK2 G2019S mutation caused ER stress and apoptosis in astrocytes [169] by impairing the sarco/ER Ca^2+^-ATPase, resulting in reduced ER Ca^2+^ levels. It also increased ER-mitochondria interaction, resulting in mitochondrial dysfunction [169]. This suggests that LRRK2 mutations increase the susceptibility of astrocytes to ER stress and mitochondrial dysfunction. Importantly, there was no significant effect on ER stress in LRRK2 G2019S neurons, but wild-type neurons co-cultured with LRRK2 G2019S astrocytes sustained more neuronal injury after α-synuclein treatment [169]. These results indicate that ER stress in astrocytes and neurons is differently affected by LRRK2 G2019S mutation, highlighting the role of astrocytic LRRK2 G2019S mutation and ER stress contributing to PD development. Moreover, astrocytic ER stress increases the production of proinflammatory cytokines and chemokines and also activates inflammatory microglia via paracrine signaling [169], indicating that astrocytic ER stress induces inflammatory astrocytic dysfunction and triggers adjacent microglial activation, which may lead to neuronal injury.

### 3.7. The Role of Astrocytic Apoptosis in PD Development

Astrocytic apoptosis could cause harmful damage to the surrounding neurons by releasing toxic astrocytic components and proinflammatory factors [170]. Astrocytic apoptosis can also impair astrocyte-neuron networks and destabilize synaptic neurotransmission, thus contributing to neuronal cell death. Prolonged astrocytic dysfunction and degeneration accelerated neuronal injury in the substantia nigra in a 6-OHDA PD rat model [100], indicating that astrocytic apoptosis could be an important contributor to PD pathology. Astrocytic apoptosis is caused by various cellular dysregulations, including oxidative stress, mitochondrial dysfunction, inflammation, disrupted Ca^2+^ homeostasis, membrane instability, and ER stress [168,171,172]. Rotenone causes astrocytic apoptosis, at least in part, by reducing astrocytic connexin 43 and increasing membrane and gap junction permeability [173]. Given that dysregulated gap junctions play an important role in neurological disorders, such as AD, HD, ischemia, and PD [173,174,175], and that connexins are critical in maintaining gap junctions to allow direct intercellular communication between adjacent cells [176], rotenone-induced dysfunctional gap junctions in astrocytes may cause the leakage of toxic substances to adjacent neural cells, including dopaminergic neurons. Astrocytic activation can lead to astrocyte apoptosis and stimulate microglial activation via cytokine and chemokine communication, indicating that the activation of astrocytes and microglia are closely connected to each other [177]. Major histocompatibility complex II levels were increased and mainly found in midbrain astrocytes and microglia rather than in neurons of the PD MPTP mouse model [178]. In addition, astrocytic apoptosis diminishes astrocyte-induced neuroprotection and antioxidant support to neurons, increasing neuronal susceptibility to toxic insults. These indicate that inhibiting astrocytic apoptosis might be an important therapeutic target to provide neuroprotection against PD insults.

## 4. Conclusions

**Figure 1:**
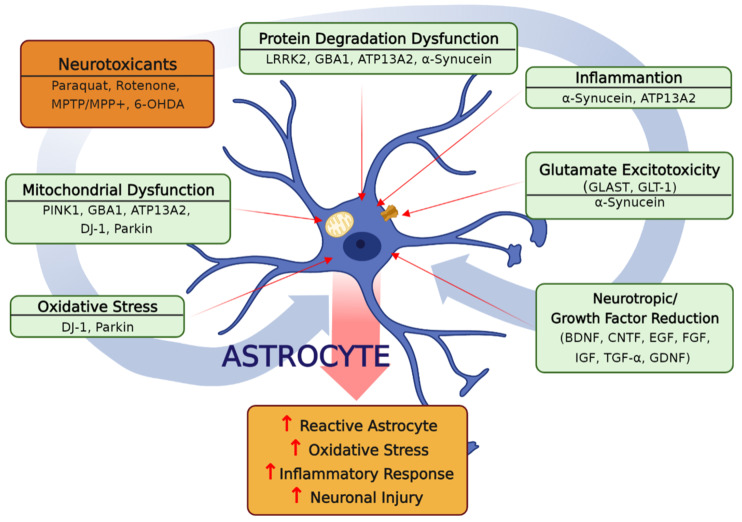
A summary of the astrocytic dysregulations contributing to dopaminergic neurodegeneration in PD gene mutations and PD neurotoxicants. Dysregulations in astrocytic mechanisms, such as oxidative stress, impairment of autophagy/mitophagy, inflammation, neurotrophic/growth factor reduction, and excitotoxicity, contribute to dopaminergic neuronal injury in PD. Several PD-mutated genes, including DJ-1, α-synuclein, LRRK2, PINK1, parkin, and PD-inducing experimental neurotoxicants, such as rotenone, MPTP/MPP^+^, paraquat, and 6-OHDA, have been shown to modulate these molecular mechanisms in astrocytes, which could exacerbate PD pathology.

In summary, astrocytes are essential neural cell types that protect neurons against PD-inducing insults. Healthy astrocytes exert their neuroprotective effects by releasing neurotrophic factors, producing antioxidants, reducing proinflammatory cytokines, and removing toxic aggregates such as α-synuclein and damaged mitochondria. However, astrocytic dysfunctions, such as oxidative stress, inflammation, autophagy impairment, and apoptosis, are commonly observed in pathological conditions in gene mutations associated with PD, such as DJ-1, α-synuclein, LRRK2, PINK1, and parkin, as well as experimental PD models using toxicants, such as paraquat, rotenone, MPTP/MPP+, and 6-OHDA (Figure 1). Understanding astrocytes’ cellular and molecular responses in these PD models will greatly contribute to the development of PD therapeutics targeting astrocytes. In addition, the findings suggest that complex pathways are involved in PD pathology, and thus, a combination of approaches may be required for therapeutic intervention.

## Figures and Tables

**Table 1:** Astrocytes contribute to PD pathology in a variety of PD mutants.

Gene	Protein	Function	Mode of Inheritance	Time of Onset *	References
SNCA	α-synuclein	Glutamate transport, inflammatory response	Autosomal dominant	Early	[5,6,7]
PARK2	Parkin	Inflammatory response, mitochondrial function	Autosomal recessive	Early	[8,9]
PARK6	PINK1	Mitochondrial function	Autosomal recessive	Early	[10]
PARK7	DJ-1	Glutamate uptake, inflammatory response, mitochondrial function	Autosomal recessive	Early	[11,12,13]
PARK8	LRRK2	Autophagy, Lysosome function	Autosomal dominant	Late	[14,15]
PARK9	ATP13A2	Inflammatory response, Lysosome function	Autosomal recessive	Early	[16]
GBA	GCase (Glucocerebrosidase)	Autophagy, Lysosome function	Autosomal recessive	Late	[17]

* Early onset, ages 21–50; late-onset, ages older than 50.

## References

[1] Lieberman A., Dziatolowski M., Gopinathan G., Kupersmith M., Neophytides A., Korein J. (1980). Evaluation of Parkinson’s disease. Adv. Biochem. Psychopharmacol..

[2] Yang W., Hamilton J.L., Kopil C., Beck J.C., Tanner C.M., Albin R.L., Dorsey E.R., Dahodwala N., Cintina I., Hogan P. (2020). Current and projected future economic burden of Parkinson’s disease in the U.S. NPJ Park. Dis..

[3] Shadrina M., Slominsky P. (2021). Modeling Parkinson’s Disease: Not Only Rodents?. Front. Aging Neurosci..

[4] Bove J., Prou D., Perier C., Przedborski S. (2005). Toxin-induced models of Parkinson’s disease. NeuroRx.

[5] Rannikko E.H., Weber S.S., Kahle P.J. (2015). Exogenous alpha-synuclein induces toll-like receptor 4 dependent inflammatory responses in astrocytes. BMC Neurosci..

[6] Gu X.L., Long C.X., Sun L., Xie C., Lin X., Cai H. (2010). Astrocytic expression of Parkinson’s disease-related A53T alpha-synuclein causes neurodegeneration in mice. Mol. Brain..

[7] Diniz L.P., Araujo A.P.B., Matias I., Garcia M.N., Barros-Aragão F.G., Reis R.A.D.M., Foguel D., Braga C., Figueiredo C.P., Romão L. (2020). Astrocyte glutamate transporters are increased in an early sporadic model of synucleinopathy. Neurochem. Int..

[8] Schmidt S., Linnartz B., Mendritzki S., Sczepan T., Lubbert M., Stichel C.C., Lubbert H. (2011). Genetic mouse models for Parkinson’s disease display severe pathology in glial cell mitochondria. Hum. Mol. Genet..

[9] Khasnavis S., Pahan K. (2014). Cinnamon Treatment Upregulates Neuroprotective Proteins Parkin and DJ-1 and Protects Dopaminergic Neurons in a Mouse Model of Parkinson’s Disease. J. Neuroimmune Pharmacol..

[10] Choi I., Kim J., Jeong H.K., Kim B., Jou I., Park S.M., Chen L., Kang U.J., Zhuang X., Joe E.H. (2013). PINK1 deficiency attenuates astrocyte proliferation through mitochondrial dysfunction, reduced AKT and increased p38 MAPK activation, and downregulation of EGFR. Glia.

[11] Kim K.S., Kim J.S., Park J.Y., Suh Y.H., Jou I., Joe E.H., Park S.M. (2013). DJ-1 associates with lipid rafts by palmitoylation and regulates lipid rafts-dependent endocytosis in astrocytes. Hum. Mol. Genet..

[12] Kim J.M., Cha S.H., Choi Y.R., Jou I., Joe E.H., Park S.M. (2016). DJ-1 deficiency impairs glutamate uptake into astrocytes via the regulation of flotillin-1 and caveolin-1 expression. Sci. Rep..

[13] Mullett S.J., Hinkle D.A. (2011). DJ-1 deficiency in astrocytes selectively enhances mitochondrial Complex I inhibitor-induced neurotoxicity. J. Neurochem..

[14] Manzoni C., Mamais A., Dihanich S., Abeti R., Soutar M.P.M., Plun-Favreau H., Giunti P., Tooze S.A., Bandopadhyay R., Lewis P.A. (2013). Inhibition of LRRK2 kinase activity stimulates macroautophagy. Biochim. Biophys. Acta.

[15] Henry A.G., Aghamohammadzadeh S., Samaroo H., Chen Y., Mou K., Needle E., Hirst W.D. (2015). Pathogenic LRRK2 mutations, through increased kinase activity, produce enlarged lysosomes with reduced degradative capacity and increase ATP13A2 expression. Hum. Mol. Genet..

[16] Qiao C., Yin N., Gu H.-Y., Zhu J.-L., Ding J.-H., Lu M., Hu G. (2016). *Atp13a2* Deficiency Aggravates Astrocyte-Mediated Neuroinflammation via NLRP3 Inflammasome Activation. CNS Neurosci. Ther..

[17] Ran C. (2016). Strong association between glucocerebrosidase mutations and Parkinson’s disease in Sweden. Neurobiol. Aging.

[18] Canet-Aviles R.M., Wilson M.A., Miller D.W., Ahmad R., McLendon C., Bandyopadhyay S., Baptista M.J., Ringe D., Petsko G.A., Cookson M.R. (2004). The Parkinson’s disease protein DJ-1 is neuroprotective due to cysteine-sulfinic acid-driven mitochondrial localization. Proc. Natl. Acad. Sci. USA.

[19] Ahn E.H., Lei K., Kang S.S., Wang Z.H., Liu X., Hong W., Wang Y.T., Edgington-Mitchell L.E., Jin L., Ye K. (2021). Mitochondrial dysfunction triggers the pathogenesis of Parkinson’s disease in neuronal C/EBPbeta transgenic mice. Mol. Psychiatry.

[20] Pajares M., Rojo A.I., Manda G., Bosca L., Cuadrado A. (2020). Inflammation in Parkinson’s Disease: Mechanisms and Therapeutic Implications. Cells.

[21] Wang B., Abraham N., Gao G., Yang Q. (2016). Dysregulation of autophagy and mitochondrial function in Parkinson’s disease. Transl. Neurodegener..

[22] Liu J., Liu W., Li R., Yang H. (2019). Mitophagy in Parkinson’s Disease: From Pathogenesis to Treatment. Cells.

[23] Alexander G.E. (2004). Biology of Parkinson’s disease: Pathogenesis and pathophysiology of a multisystem neurodegenerative disorder. Dialogues Clin. Neurosci..

[24] Kettenmann H., Verkhratsky A. (2008). Neuroglia: The 150 years after. Trends Neurosci..

[25] Jakel S., Dimou L. (2017). Glial Cells and Their Function in the Adult Brain: A Journey through the History of Their Ablation. Front. Cell. Neurosci..

[26] Halliday G.M., Stevens C.H. (2011). Glia: Initiators and progressors of pathology in Parkinson’s disease. Mov. Disord..

[27] Verkhratsky A., Nedergaard M., Hertz L. (2015). Why are astrocytes important?. Neurochem Res..

[28] Bonifati V., Rizzu P., van Baren M.J., Schaap O., Breedveld G.J., Krieger E., Dekker M.C., Squitieri F., Ibanez P., Joosse M. (2003). Mutations in the DJ-1 gene associated with autosomal recessive early-onset parkinsonism. Science.

[29] Honbou K., Suzuki N.N., Horiuchi M., Niki T., Taira T., Ariga H., Inagaki F. (2003). The crystal structure of DJ-1, a protein related to male fertility and Parkinson’s disease. J. Biol. Chem..

[30] Ariga H., Takahashi-Niki K., Kato I., Maita H., Niki T., Iguchi-Ariga S.M. (2013). Neuroprotective function of DJ-1 in Parkinson’s disease. Oxid. Med. Cell. Longev..

[31] Bandopadhyay R., Kingsbury A.E., Cookson M.R., Reid A.R., Evans I.M., Hope A.D., Pittman A.M., Lashley T., Canet-Aviles R., Miller D.W. (2004). The expression of DJ-1 (PARK7) in normal human CNS and idiopathic Parkinson’s disease. Brain.

[32] Mullett S.J., Hamilton R.L., Hinkle D.A. (2009). DJ-1 immunoreactivity in human brain astrocytes is dependent on infarct presence and infarct age. Neuropathology.

[33] Simons K., Ehehalt R. (2002). Cholesterol, lipid rafts, and disease. J. Clin. Invest..

[34] Butchbach M.E., Tian G., Guo H., Lin C.L. (2004). Association of excitatory amino acid transporters, especially EAAT2, with cholesterol-rich lipid raft microdomains: Importance for excitatory amino acid transporter localization and function. J. Biol. Chem..

[35] Roberts P.J., Davies S.W. (1987). Excitatory receptors and their role in excitotoxicity. Biochem. Soc. Trans..

[36] Ashley A.K., Hinds A.I., Hanneman W.H., Tjalkens R.B., Legare M.E. (2016). DJ-1 mutation decreases astroglial release of inflammatory mediators. Neurotoxicology.

[37] Mullett S.J., Hinkle D.A. (2009). DJ-1 knock-down in astrocytes impairs astrocyte-mediated neuroprotection against rotenone. Neurobiol. Dis..

[38] Lev N., Barhum Y., Ben-Zur T., Melamed E., Steiner I., Offen D. (2013). Knocking out DJ-1 attenuates astrocytes neuroprotection against 6-hydroxydopamine toxicity. J. Mol. Neurosci..

[39] Larsen N.J., Ambrosi G., Mullett S.J., Berman S.B., Hinkle D.A. (2011). DJ-1 knock-down impairs astrocyte mitochondrial function. Neuroscience.

[40] Bantle C.M., Hirst W.D., Weihofen A., Shlevkov E. (2020). Mitochondrial Dysfunction in Astrocytes: A Role in Parkinson’s Disease?. Front. Cell. Dev. Biol..

[41] Peng L., Zhao Y., Li Y., Zhou Y., Li L., Lei S., Yu S., Zhao Y. (2019). Effect of DJ-1 on the neuroprotection of astrocytes subjected to cerebral ischemia/reperfusion injury. J. Mol. Med..

[42] Thomas K.J., McCoy M.K., Blackinton J., Beilina A., van der Brug M., Sandebring A., Miller D., Maric D., Cedazo-Minguez A., Cookson M.R. (2011). DJ-1 acts in parallel to the PINK1/parkin pathway to control mitochondrial function and autophagy. Hum. Mol. Genet..

[43] Dolgacheva L.P., Berezhnov A.V., Fedotova E.I., Zinchenko V.P., Abramov A.Y. (2019). Role of DJ-1 in the mechanism of pathogenesis of Parkinson’s disease. J. Bioenerg. Biomembr..

[44] Bjorkblom B., Adilbayeva A., Maple-Grodem J., Piston D., Okvist M., Xu X.M., Brede C., Larsen J.P., Moller S.G. (2013). Parkinson disease protein DJ-1 binds metals and protects against metal-induced cytotoxicity. J. Biol. Chem..

[45] Waak J., Weber S.S., Waldenmaier A., Gorner K., Alunni-Fabbroni M., Schell H., Vogt-Weisenhorn D., Pham T.T., Reumers V., Baekelandt V. (2009). Regulation of astrocyte inflammatory responses by the Parkinson’s disease-associated gene DJ-1. FASEB J..

[46] Choi D.J., Yang H., Gaire S., Lee K.A., An J., Kim B.G., Jou I., Park S.M., Joe E.H. (2020). Critical roles of astrocytic-CCL2-dependent monocyte infiltration in a DJ-1 knockout mouse model of delayed brain repair. Glia.

[47] Zhang G., Xia Y., Wan F., Ma K., Guo X., Kou L., Yin S., Han C., Liu L., Huang J. (2018). New Perspectives on Roles of Alpha-Synuclein in Parkinson’s Disease. Front. Aging Neurosci..

[48] Bonini N.M., Giasson B.I. (2005). Snaring the function of alpha-synuclein. Cell.

[49] Moore S.A., Yoder E., Murphy S., Dutton G.R., Spector A.A. (1991). Astrocytes, not neurons, produce docosahexaenoic acid (22:6 omega-3) and arachidonic acid (20:4 omega-6). J. Neurochem..

[50] Solano S.M., Miller D.W., Augood S.J., Young A.B., Penney J.B. (2000). Expression of alpha-synuclein, parkin, and ubiquitin carboxy-terminal hydrolase L1 mRNA in human brain: Genes associated with familial Parkinson’s disease. Ann. Neurol..

[51] Alecu I., Bennett S.A.L. (2019). Dysregulated Lipid Metabolism and Its Role in alpha-Synucleinopathy in Parkinson’s Disease. Front. Neurosci..

[52] Booth H.D.E., Hirst W.D., Wade-Martins R. (2017). The Role of Astrocyte Dysfunction in Parkinson’s Disease Pathogenesis. Trends Neurosci..

[53] Castagnet P.I., Golovko M.Y., Barcelo-Coblijn G.C., Nussbaum R.L., Murphy E.J. (2005). Fatty acid incorporation is decreased in astrocytes cultured from alpha-synuclein gene-ablated mice. J. Neurochem..

[54] Braak H., Sastre M., Del Tredici K. (2007). Development of alpha-synuclein immunoreactive astrocytes in the forebrain parallels stages of intraneuronal pathology in sporadic Parkinson’s disease. Acta Neuropathol..

[55] Braidy N., Gai W.P., Xu Y.H., Sachdev P., Guillemin G.J., Jiang X.M., Ballard J.W., Horan M.P., Fang Z.M., Chong B.H. (2013). Uptake and mitochondrial dysfunction of alpha-synuclein in human astrocytes, cortical neurons and fibroblasts. Transl. Neurodegener..

[56] Lee H.J., Suk J.E., Patrick C., Bae E.J., Cho J.H., Rho S., Hwang D., Masliah E., Lee S.J. (2010). Direct transfer of alpha-synuclein from neuron to astroglia causes inflammatory responses in synucleinopathies. J. Biol. Chem..

[57] Yang Q., Wang Y., Zhao C., Pang S., Lu J., Chan P. (2022). Alpha-Synuclein aggregation causes muscle atrophy through neuromuscular junction degeneration. J. Cachexia Sarcopenia Muscle.

[58] Tsunemi T., Ishiguro Y., Yoroisaka A., Valdez C., Miyamoto K., Ishikawa K., Saiki S., Akamatsu W., Hattori N., Krainc D. (2020). Astrocytes Protect Human Dopaminergic Neurons from alpha-Synuclein Accumulation and Propagation. J. Neurosci..

[59] Ramirez A., Heimbach A., Grundemann J., Stiller B., Hampshire D., Cid L.P., Goebel I., Mubaidin A.F., Wriekat A.L., Roeper J. (2006). Hereditary parkinsonism with dementia is caused by mutations in ATP13A2, encoding a lysosomal type 5 P-type ATPase. Nat. Genet..

[60] Park J.S., Blair N.F., Sue C.M. (2015). The role of ATP13A2 in Parkinson’s disease: Clinical phenotypes and molecular mechanisms. Mov. Disord..

[61] Dang T., Cao W.J., Zhao R., Lu M., Hu G., Qiao C. (2022). ATP13A2 protects dopaminergic neurons in Parkinson’s disease: From biology to pathology. J. Biomed. Res..

[62] Zimprich A., Biskup S., Leitner P., Lichtner P., Farrer M., Lincoln S., Kachergus J., Hulihan M., Uitti R.J., Calne D.B. (2004). Mutations in LRRK2 cause autosomal-dominant parkinsonism with pleomorphic pathology. Neuron.

[63] Paisan-Ruiz C., Jain S., Evans E.W., Gilks W.P., Simon J., van der Brug M., Lopez de Munain A., Aparicio S., Gil A.M., Khan N. (2004). Cloning of the gene containing mutations that cause PARK8-linked Parkinson’s disease. Neuron.

[64] Herbst S., Gutierrez M.G. (2019). LRRK2 in Infection: Friend or Foe?. ACS Infect. Dis..

[65] Wallings R.L., Tansey M.G. (2019). LRRK2 regulation of immune-pathways and inflammatory disease. Biochem. Soc. Trans..

[66] Madureira M., Connor-Robson N., Wade-Martins R. (2020). LRRK2: Autophagy and Lysosomal Activity. Front. Neurosci..

[67] Giasson B.I., Van Deerlin V.M. (2008). Mutations in LRRK2 as a cause of Parkinson’s disease. Neurosignals.

[68] Greggio E., Jain S., Kingsbury A., Bandopadhyay R., Lewis P., Kaganovich A., van der Brug M.P., Beilina A., Blackinton J., Thomas K.J. (2006). Kinase activity is required for the toxic effects of mutant LRRK2/dardarin. Neurobiol. Dis..

[69] Di Maio R., Hoffman E.K., Rocha E.M., Keeney M.T., Sanders L.H., De Miranda B.R., Zharikov A., Van Laar A., Stepan A.F., Lanz T.A. (2018). LRRK2 activation in idiopathic Parkinson’s disease. Sci. Trans. Med..

[70] Miklossy J., Arai T., Guo J.P., Klegeris A., Yu S., McGeer E.G., McGeer P.L. (2006). LRRK2 expression in normal and pathologic human brain and in human cell lines. J. Neuropathol. Exp. Neurol..

[71] di Domenico A., Carola G., Calatayud C., Pons-Espinal M., Munoz J.P., Richaud-Patin Y., Fernandez-Carasa I., Gut M., Faella A., Parameswaran J. (2019). Patient-Specific iPSC-Derived Astrocytes Contribute to Non-Cell-Autonomous Neurodegeneration in Parkinson’s Disease. Stem Cell Rep..

[72] Streubel-Gallasch L., Giusti V., Sandre M., Tessari I., Plotegher N., Giusto E., Masato A., Iovino L., Battisti I., Arrigoni G. (2021). Parkinson’s Disease-Associated LRRK2 Interferes with Astrocyte-Mediated Alpha-Synuclein Clearance. Mol. Neurobiol..

[73] Pickles S., Vigie P., Youle R.J. (2018). Mitophagy and Quality Control Mechanisms in Mitochondrial Maintenance. Curr. Biol..

[74] Kawajiri S., Saiki S., Sato S., Hattori N. (2011). Genetic mutations and functions of PINK1. Trends Pharmacol. Sci..

[75] Barodia S.K., McMeekin L.J., Creed R.B., Quinones E.K., Cowell R.M., Goldberg M.S. (2019). PINK1 phosphorylates ubiquitin predominantly in astrocytes. NPJ Park. Dis..

[76] Sun L., Shen R., Agnihotri S.K., Chen Y., Huang Z., Bueler H. (2018). Lack of PINK1 alters glia innate immune responses and enhances inflammation-induced, nitric oxide-mediated neuron death. Sci. Rep..

[77] Choi I., Choi D.J., Yang H., Woo J.H., Chang M.Y., Kim J.Y., Sun W., Park S.M., Jou I., Lee S.H. (2016). PINK1 expression increases during brain development and stem cell differentiation, and affects the development of GFAP-positive astrocytes. Mol. Brain.

[78] Olszewska D.A., Lynch T. (2015). Will crystal parkin help in understanding the future of Parkinson’s disease?. Front. Neurol..

[79] Ledesma M.D., Galvan C., Hellias B., Dotti C., Jensen P.H. (2002). Astrocytic but not neuronal increased expression and redistribution of parkin during unfolded protein stress. J. Neurochem..

[80] Russ K., Teku G., Bousset L., Redeker V., Piel S., Savchenko E., Pomeshchik Y., Savistchenko J., Stummann T.C., Azevedo C. (2021). TNF-alpha and alpha-synuclein fibrils differently regulate human astrocyte immune reactivity and impair mitochondrial respiration. Cell Rep..

[81] Pickrell A.M., Youle R.J. (2015). The roles of PINK1, parkin, and mitochondrial fidelity in Parkinson’s disease. Neuron.

[82] Davis C.H., Kim K.Y., Bushong E.A., Mills E.A., Boassa D., Shih T., Kinebuchi M., Phan S., Zhou Y., Bihlmeyer N.A. (2014). Transcellular degradation of axonal mitochondria. Proc. Natl. Acad. Sci. USA.

[83] Morales I., Sanchez A., Puertas-Avendano R., Rodriguez-Sabate C., Perez-Barreto A., Rodriguez M. (2020). Neuroglial transmitophagy and Parkinson’s disease. Glia.

[84] Youle R.J. (2019). Mitochondria-Striking a balance between host and endosymbiont. Science.

[85] Gkikas I., Palikaras K., Tavernarakis N. (2018). The Role of Mitophagy in Innate Immunity. Front. Immunol..

[86] Iyer S.S., He Q., Janczy J.R., Elliott E.I., Zhong Z., Olivier A.K., Sadler J.J., Knepper-Adrian V., Han R., Qiao L. (2013). Mitochondrial cardiolipin is required for Nlrp3 inflammasome activation. Immunity.

[87] Kahns S., Kalai M., Jakobsen L.D., Clark B.F., Vandenabeele P., Jensen P.H. (2003). Caspase-1 and caspase-8 cleave and inactivate cellular parkin. J. Biol. Chem..

[88] Yu M., Zhang K., Qi W., Huang Z., Ye J., Ma Y., Liao M., Ning Z. (2014). Expression pattern of NLRP3 and its related cytokines in the lung and brain of avian influenza virus H9N2 infected BALB/c mice. Virol. J..

[89] Neumann J., Bras J., Deas E., O’Sullivan S.S., Parkkinen L., Lachmann R.H., Li A., Holton J., Guerreiro R., Paudel R. (2009). Glucocerebrosidase mutations in clinical and pathologically proven Parkinson’s disease. Brain.

[90] Cahoy J.D., Emery B., Kaushal A., Foo L.C., Zamanian J.L., Christopherson K.S., Xing Y., Lubischer J.L., Krieg P.A., Krupenko S.A. (2008). A transcriptome database for astrocytes, neurons, and oligodendrocytes: A new resource for understanding brain development and function. J. Neurosci..

[91] Aflaki E., Stubblefield B.K., McGlinchey R.P., McMahon B., Ory D.S., Sidransky E. (2020). A characterization of Gaucher iPS-derived astrocytes: Potential implications for Parkinson’s disease. Neurobiol. Dis..

[92] Osellame L.D., Rahim A.A., Hargreaves I.P., Gegg M.E., Richard-Londt A., Brandner S., Waddington S.N., Schapira A.H.V., Duchen M.R. (2013). Mitochondria and quality control defects in a mouse model of Gaucher disease--links to Parkinson’s disease. Cell Metab..

[93] Osellame L.D., Duchen M.R. (2013). Defective quality control mechanisms and accumulation of damaged mitochondria link Gaucher and Parkinson diseases. Autophagy.

[94] Sanyal A., DeAndrade M.P., Novis H.S., Lin S., Chang J., Lengacher N., Tomlinson J.J., Tansey M.G., LaVoie M.J. (2020). Lysosome and Inflammatory Defects in GBA1-Mutant Astrocytes Are Normalized by LRRK2 Inhibition. Mov. Disord..

[95] Farfel-Becker T., Vitner E.B., Kelly S.L., Bame J.R., Duan J., Shinder V., Merrill A.H., Dobrenis K., Futerman A.H. (2014). Neuronal accumulation of glucosylceramide in a mouse model of neuronopathic Gaucher disease leads to neurodegeneration. Hum. Mol. Genet..

[96] Ginns E.I., Mak S.K., Ko N., Karlgren J., Akbarian S., Chou V.P., Guo Y., Lim A., Samuelsson S., LaMarca M.L. (2014). Neuroinflammation and alpha-synuclein accumulation in response to glucocerebrosidase deficiency are accompanied by synaptic dysfunction. Mol. Genet. Metab..

[97] Goker-Alpan O., Giasson B.I., Eblan M.J., Nguyen J., Hurtig H.I., Lee V.M., Trojanowski J.Q., Sidransky E. (2006). Glucocerebrosidase mutations are an important risk factor for Lewy body disorders. Neurology.

[98] Talifu Z., Liu J.Y., Pan Y.Z., Ke H., Zhang C.J., Xu X., Gao F., Yu Y., Du L.J., Li J.J. (2023). In vivo astrocyte-to-neuron reprogramming for central nervous system regeneration: A narrative review. Neural. Regen. Res..

[99] Wei Z.D., Shetty A.K. (2021). Treating Parkinson’s disease by astrocyte reprogramming: Progress and challenges. Sci. Adv..

[100] Kuter K., Olech L., Glowacka U. (2018). Prolonged Dysfunction of Astrocytes and Activation of Microglia Accelerate Degeneration of Dopaminergic Neurons in the Rat Substantia Nigra and Block Compensation of Early Motor Dysfunction Induced by 6-OHDA. Mol. Neurobiol..

[101] Kato H., Araki T., Imai Y., Takahashi A., Itoyama Y. (2003). Protection of dopaminergic neurons with a novel astrocyte modulating agent (R)-(−)-2-propyloctanoic acid (ONO-2506) in an MPTP-mouse model of Parkinson’s disease. J. Neurol. Sci..

[102] Himeda T., Kadoguchi N., Kamiyama Y., Kato H., Maegawa H., Araki T. (2006). Neuroprotective effect of arundic acid, an astrocyte-modulating agent, in mouse brain against MPTP (1-methyl-4-phenyl-1,2,3,6-tetrahydropyridine) neurotoxicity. Neuropharmacology.

[103] Wei Z., Li X., Li X., Liu Q., Cheng Y. (2018). Oxidative Stress in Parkinson’s Disease: A Systematic Review and Meta-Analysis. Front. Mol. Neurosci..

[104] Asanuma M., Okumura-Torigoe N., Miyazaki I., Murakami S., Kitamura Y., Sendo T. (2019). Region-Specific Neuroprotective Features of Astrocytes against Oxidative Stress Induced by 6-Hydroxydopamine. Int. J. Mol. Sci..

[105] Bhatia T.N., Pant D.B., Eckhoff E.A., Gongaware R.N., Do T., Hutchison D.F., Gleixner A.M., Leak R.K. (2019). Astrocytes Do Not Forfeit Their Neuroprotective Roles After Surviving Intense Oxidative Stress. Front. Mol. Neurosci..

[106] Mullett S.J., Di Maio R., Greenamyre J.T., Hinkle D.A. (2013). DJ-1 expression modulates astrocyte-mediated protection against neuronal oxidative stress. J. Mol. Neurosci..

[107] De Miranda B.R., Rocha E.M., Bai Q., El Ayadi A., Hinkle D., Burton E.A., Timothy Greenamyre J. (2018). Astrocyte-specific DJ-1 overexpression protects against rotenone-induced neurotoxicity in a rat model of Parkinson’s disease. Neurobiol. Dis..

[108] Olesen B.T., Clausen J., Vang O. (2008). Characterization of the transcriptional profile in primary astrocytes after oxidative stress induced by Paraquat. Neurotoxicology.

[109] Pistollato F., Canovas-Jorda D., Zagoura D., Bal-Price A. (2017). Nrf2 pathway activation upon rotenone treatment in human iPSC-derived neural stem cells undergoing differentiation towards neurons and astrocytes. Neurochem. Int..

[110] Sundar Boyalla S., Barbara Victor M., Roemgens A., Beyer C., Arnold S. (2011). Sex- and brain region-specific role of cytochrome c oxidase in 1-methyl-4-phenylpyridinium-mediated astrocyte vulnerability. J. Neurosci. Res..

[111] Swarnkar S., Singh S., Goswami P., Mathur R., Patro I.K., Nath C. (2012). Astrocyte activation: A key step in rotenone induced cytotoxicity and DNA damage. Neurochem. Res..

[112] Rathinam M.L., Watts L.T., Narasimhan M., Riar A.K., Mahimainathan L., Henderson G.I. (2012). Astrocyte mediated protection of fetal cerebral cortical neurons from rotenone and paraquat. Environ. Toxicol. Pharmacol..

[113] Chen P.C., Vargas M.R., Pani A.K., Smeyne R.J., Johnson D.A., Kan Y.W., Johnson J.A. (2009). Nrf2-mediated neuroprotection in the MPTP mouse model of Parkinson’s disease: Critical role for the astrocyte. Proc. Natl. Acad. Sci. USA.

[114] Guo Q., Wang B., Wang X., Smith W.W., Zhu Y., Liu Z. (2021). Activation of Nrf2 in Astrocytes Suppressed PD-Like Phenotypes via Antioxidant and Autophagy Pathways in Rat and Drosophila Models. Cells.

[115] Clements C.M., McNally R.S., Conti B.J., Mak T.W., Ting J.P. (2006). DJ-1, a cancer- and Parkinson’s disease-associated protein, stabilizes the antioxidant transcriptional master regulator Nrf2. Proc. Natl. Acad. Sci. USA.

[116] Kim H.S., Son T.G., Park H.R., Lee Y., Jung Y., Ishigami A., Lee J. (2013). Senescence marker protein 30 deficiency increases Parkinson’s pathology by impairing astrocyte activation. Neurobiol. Aging.

[117] McNaught K.S., Jenner P. (1999). Altered glial function causes neuronal death and increases neuronal susceptibility to 1-methyl-4-phenylpyridinium- and 6-hydroxydopamine-induced toxicity in astrocytic/ventral mesencephalic co-cultures. J. Neurochem..

[118] McGeer P.L., Itagaki S., Boyes B.E., McGeer E.G. (1988). Reactive microglia are positive for HLA-DR in the substantia nigra of Parkinson’s and Alzheimer’s disease brains. Neurology.

[119] Long-Smith C.M., Sullivan A.M., Nolan Y.M. (2009). The influence of microglia on the pathogenesis of Parkinson’s disease. Prog. Neurobiol..

[120] Sun H., Liang R., Yang B., Zhou Y., Liu M., Fang F., Ding J., Fan Y., Hu G. (2016). Aquaporin-4 mediates communication between astrocyte and microglia: Implications of neuroinflammation in experimental Parkinson’s disease. Neuroscience.

[121] Yang Y.J., Zhang S., Ding J.H., Zhou F., Hu G. (2009). Iptakalim protects against MPP+-induced degeneration of dopaminergic neurons in association with astrocyte activation. Int. J. Neuropsychopharmacol..

[122] Zhang J.X., Zhou K.G., Yin Y.X., Jin L.J., Tong W.F., Guo J., Yu L.H., Ye X.C., Jiang M. (2022). Mesencephalic astrocyte-derived neurotrophic factor (MANF) prevents the neuroinflammation induced dopaminergic neurodegeneration. Exp. Gerontol..

[123] Michalovicz L.T., Kelly K.A., Vashishtha S., Ben-Hamo R., Efroni S., Miller J.V., Locker A.R., Sullivan K., Broderick G., Miller D.B. (2019). Astrocyte-specific transcriptome analysis using the ALDH1L1 bacTRAP mouse reveals novel biomarkers of astrogliosis in response to neurotoxicity. J. Neurochem..

[124] Mori T., Town T., Tan J., Tateishi N., Asano T. (2005). Modulation of Astrocytic Activation by Arundic Acid (ONO-2506) Mitigates Detrimental Effects of the Apolipoprotein E4 Isoform after Permanent Focal Ischemia in Apolipoprotein E Knock-in Mice. J. Cereb. Blood Flow Metab..

[125] Kato H., Kurosaki R., Oki C., Araki T. (2004). Arundic acid, an astrocyte-modulating agent, protects dopaminergic neurons against MPTP neurotoxicity in mice. Brain Res..

[126] Wang H.M., Zhang T., Li Q., Huang J.K., Chen R.F., Sun X.J. (2013). Inhibition of glycogen synthase kinase-3beta by lithium chloride suppresses 6-hydroxydopamine-induced inflammatory response in primary cultured astrocytes. Neurochem. Int..

[127] Gupta S., Goswami P., Biswas J., Joshi N., Sharma S., Nath C., Singh S. (2015). 6-Hydroxydopamine and lipopolysaccharides induced DNA damage in astrocytes: Involvement of nitric oxide and mitochondria. Mutat. Res. Genet. Toxicol. Environ. Mutagen..

[128] Zhang J., Culp M.L., Craver J.G., Darley-Usmar V. (2018). Mitochondrial function and autophagy: Integrating proteotoxic, redox, and metabolic stress in Parkinson’s disease. J. Neurochem..

[129] Mebratu Y.A., Negasi Z.H., Dutta S., Rojas-Quintero J., Tesfaigzi Y. (2020). Adaptation of Proteasomes and Lysosomes to Cellular Environments. Cells.

[130] Lee H.J., Patel S., Lee S.J. (2005). Intravesicular localization and exocytosis of alpha-synuclein and its aggregates. J. Neurosci..

[131] Deleidi M., Maetzler W. (2012). Protein clearance mechanisms of alpha-synuclein and amyloid-Beta in lewy body disorders. Int. J. Alzheimers Dis..

[132] Morales I., Sanchez A., Rodriguez-Sabate C., Rodriguez M. (2017). Striatal astrocytes engulf dopaminergic debris in Parkinson’s disease: A study in an animal model. PLoS ONE.

[133] Cavaliere F., Cerf L., Dehay B., Ramos-Gonzalez P., De Giorgi F., Bourdenx M., Bessede A., Obeso J.A., Matute C., Ichas F. (2017). In vitro alpha-synuclein neurotoxicity and spreading among neurons and astrocytes using Lewy body extracts from Parkinson disease brains. Neurobiol. Dis..

[134] Morales I., Puertas-Avendano R., Sanchez A., Perez-Barreto A., Rodriguez-Sabate C., Rodriguez M. (2021). Astrocytes and retrograde degeneration of nigrostriatal dopaminergic neurons in Parkinson’s disease: Removing axonal debris. Transl. Neurodegener..

[135] Janda E., Lascala A., Carresi C., Parafati M., Aprigliano S., Russo V., Savoia C., Ziviani E., Musolino V., Morani F. (2015). Parkinsonian toxin-induced oxidative stress inhibits basal autophagy in astrocytes via NQO2/quinone oxidoreductase 2: Implications for neuroprotection. Autophagy.

[136] Zhao Z.H., Chen Z.T., Zhou R.L., Zhang X., Ye Q.Y., Wang Y.Z. (2018). Increased DJ-1 and alpha-Synuclein in Plasma Neural-Derived Exosomes as Potential Markers for Parkinson’s Disease. Front. Aging Neurosci..

[137] Rostami J., Holmqvist S., Lindstrom V., Sigvardson J., Westermark G.T., Ingelsson M., Bergstrom J., Roybon L., Erlandsson A. (2017). Human Astrocytes Transfer Aggregated Alpha-Synuclein via Tunneling Nanotubes. J. Neurosci..

[138] van Veen S., Sorensen D.M., Holemans T., Holen H.W., Palmgren M.G., Vangheluwe P. (2014). Cellular function and pathological role of ATP13A2 and related P-type transport ATPases in Parkinson’s disease and other neurological disorders. Front. Mol. Neurosci..

[139] Tsunemi T., Krainc D. (2014). Zn(2)(+) dyshomeostasis caused by loss of ATP13A2/PARK9 leads to lysosomal dysfunction and alpha-synuclein accumulation. Hum. Mol. Genet..

[140] Tsunemi T., Perez-Rosello T., Ishiguro Y., Yoroisaka A., Jeon S., Hamada K., Rammonhan M., Wong Y.C., Xie Z., Akamatsu W. (2019). Increased Lysosomal Exocytosis Induced by Lysosomal Ca(2+) Channel Agonists Protects Human Dopaminergic Neurons from alpha-Synuclein Toxicity. J. Neurosci..

[141] Rocha S.M., Bantle C.M., Aboellail T., Chatterjee D., Smeyne R.J., Tjalkens R.B. (2022). Rotenone induces regionally distinct alpha-synuclein protein aggregation and activation of glia prior to loss of dopaminergic neurons in C57Bl/6 mice. Neurobiol. Dis..

[142] Wang R., Zhao X., Xu J., Wen Y., Li A., Lu M., Zhou J. (2018). Astrocytic JWA deletion exacerbates dopaminergic neurodegeneration by decreasing glutamate transporters in mice. Cell Death Dis..

[143] Loschmann P.A., De Groote C., Smith L., Wullner U., Fischer G., Kemp J.A., Jenner P., Klockgether T. (2004). Antiparkinsonian activity of Ro 25-6981, a NR2B subunit specific NMDA receptor antagonist, in animal models of Parkinson’s disease. Exp. Neurol..

[144] Ferrarese C., Tremolizzo L., Rigoldi M., Sala G., Begni B., Brighina L., Ricci G., Albizzati M.G., Piolti R., Crosti F. (2001). Decreased platelet glutamate uptake and genetic risk factors in patients with Parkinson’s disease. Neurol. Sci..

[145] Iovino L., Giusti V., Pischedda F., Giusto E., Plotegher N., Marte A., Battisti I., Di Iacovo A., Marku A., Piccoli G. (2022). Trafficking of the glutamate transporter is impaired in LRRK2-related Parkinson’s disease. Acta Neuropathol..

[146] Zhang Y., He X., Meng X., Wu X., Tong H., Zhang X., Qu S. (2017). Regulation of glutamate transporter trafficking by Nedd4-2 in a Parkinson’s disease model. Cell Death Dis..

[147] Chung E.K., Chen L.W., Chan Y.S., Yung K.K. (2008). Downregulation of glial glutamate transporters after dopamine denervation in the striatum of 6-hydroxydopamine-lesioned rats. J. Comp. Neurol..

[148] Wei L., Chen C., Ding L., Mo M., Zou J., Lu Z., Li H., Wu H., Dai Y., Xu P. (2019). Wnt1 Promotes EAAT2 Expression and Mediates the Protective Effects of Astrocytes on Dopaminergic Cells in Parkinson’s Disease. Neural. Plast..

[149] Xiao N., Le Q.T. (2016). Neurotrophic Factors and Their Potential Applications in Tissue Regeneration. Arch. Immunol. Et Ther. Exp..

[150] Mogi M., Togari A., Kondo T., Mizuno Y., Komure O., Kuno S., Ichinose H., Nagatsu T. (1999). Brain-derived growth factor and nerve growth factor concentrations are decreased in the substantia nigra in Parkinson’s disease. Neurosci. Lett..

[151] Howells D.W. (2000). Reduced BDNF mRNA expression in the Parkinson’s disease substantia nigra. Exp. Neurol..

[152] Du F., Li R., Huang Y., Li X., Le W. (2005). Dopamine D3 receptor-preferring agonists induce neurotrophic effects on mesencephalic dopamine neurons. Eur. J. Neurosci..

[153] Streifel K.M., Gonzales A.L., De Miranda B., Mouneimne R., Earley S., Tjalkens R. (2014). Dopaminergic neurotoxicants cause biphasic inhibition of purinergic calcium signaling in astrocytes. PLoS ONE.

[154] Miyazaki I., Asanuma M. (2020). Neuron-Astrocyte Interactions in Parkinson’s Disease. Cells.

[155] Rangasamy S.B., Soderstrom K., Bakay R.A., Kordower J.H. (2010). Neurotrophic factor therapy for Parkinson’s disease. Prog. Brain Res..

[156] Yuan L.J., Zhang M., Chen S., Chen W.F. (2021). Anti-inflammatory effect of IGF-1 is mediated by IGF-1R cross talk with GPER in MPTP/MPP(+)-induced astrocyte activation. Mol. Cell Endocrinol..

[157] Datta I., Ganapathy K., Razdan R., Bhonde R. (2018). Location and Number of Astrocytes Determine Dopaminergic Neuron Survival and Function Under 6-OHDA Stress Mediated Through Differential BDNF Release. Mol. Neurobiol..

[158] Sandhu J.K., Gardaneh M., Iwasiow R., Lanthier P., Gangaraju S., Ribecco-Lutkiewicz M., Tremblay R., Kiuchi K., Sikorska M. (2009). Astrocyte-secreted GDNF and glutathione antioxidant system protect neurons against 6OHDA cytotoxicity. Neurobiol. Dis..

[159] Kordower J.H., Palfi S., Chen E.Y., Ma S.Y., Sendera T., Cochran E.J., Cochran E.J., Mufson E.J., Penn R., Goetz C.G. (1999). Clinicopathological findings following intraventricular glial-derived neurotrophic factor treatment in a patient with Parkinson’s disease. Ann. Neurol..

[160] Lang A.E., Gill S., Patel N.K., Lozano A., Nutt J.G., Penn R., Brooks D.J., Hotton G., Moro E., Heywood P. (2006). Randomized controlled trial of intraputamenal glial cell line-derived neurotrophic factor infusion in Parkinson disease. Ann. Neurol..

[161] Petrova P., Raibekas A., Pevsner J., Vigo N., Anafi M., Moore M.K., Peaire A.E., Shridhar V., Smith D.I., Kelly J. (2003). MANF: A new mesencephalic, astrocyte-derived neurotrophic factor with selectivity for dopaminergic neurons. J. Mol. Neurosci..

[162] Zhang J., Tong W., Sun H., Jiang M., Shen Y., Liu Y., Gu H., Guo J., Fang J., Jin L. (2017). Nrf2-mediated neuroprotection by MANF against 6-OHDA-induced cell damage via PI3K/AKT/GSK3beta pathway. Exp. Gerontol..

[163] Huang J., Chen C., Gu H., Li C., Fu X., Jiang M., Sun H., Xu J., Fang J., Jin L. (2016). Mesencephalic astrocyte-derived neurotrophic factor reduces cell apoptosis via upregulating GRP78 in SH-SY5Y cells. Cell. Biol. Int..

[164] Voutilainen M.H., Back S., Porsti E., Toppinen L., Lindgren L., Lindholm P., Peranen J., Saarma M., Tuominen R.K. (2009). Mesencephalic astrocyte-derived neurotrophic factor is neurorestorative in rat model of Parkinson’s disease. J. Neurosci..

[165] Estebanez B., de Paz J.A., Cuevas M.J., Gonzalez-Gallego J. (2018). Endoplasmic Reticulum Unfolded Protein Response, Aging and Exercise: An Update. Front. Physiol..

[166] Colla E., Coune P., Liu Y., Pletnikova O., Troncoso J.C., Iwatsubo T., Schneider B.L., Lee M.K. (2012). Endoplasmic reticulum stress is important for the manifestations of alpha-synucleinopathy in vivo. J. Neurosci..

[167] Colla E., Jensen P.H., Pletnikova O., Troncoso J.C., Glabe C., Lee M.K. (2012). Accumulation of toxic alpha-synuclein oligomer within endoplasmic reticulum occurs in alpha-synucleinopathy in vivo. J. Neurosci..

[168] Liu M., Qin L., Wang L., Tan J., Zhang H., Tang J., Shen X., Tan L., Wang C. (2018). Alpha-synuclein induces apoptosis of astrocytes by causing dysfunction of the endoplasmic reticulum-Golgi compartment. Mol. Med. Rep..

[169] Lee J.H., Han J.H., Kim H., Park S.M., Joe E.H., Jou I. (2019). Parkinson’s disease-associated LRRK2-G2019S mutant acts through regulation of SERCA activity to control ER stress in astrocytes. Acta Neuropathol. Commun..

[170] Takuma K., Baba A., Matsuda T. (2004). Astrocyte apoptosis: Implications for neuroprotection. Prog. Neurobiol..

[171] Liu Y., Zeng X., Hui Y., Zhu C., Wu J., Taylor D.H., Ji J., Fan W., Huang Z., Hu J. (2015). Activation of alpha7 nicotinic acetylcholine receptors protects astrocytes against oxidative stress-induced apoptosis: Implications for Parkinson’s disease. Neuropharmacology.

[172] Paquet M., Ribeiro F.M., Guadagno J., Esseltine J.L., Ferguson S.S., Cregan S.P. (2013). Role of metabotropic glutamate receptor 5 signaling and homer in oxygen glucose deprivation-mediated astrocyte apoptosis. Mol. Brain.

[173] Zhang S., Liang R., Zhou F., Huang X., Ding J.H., Hu G. (2011). Reversal of rotenone-induced dysfunction of astrocytic connexin43 by opening mitochondrial ATP-sensitive potassium channels. Cell Mol. Neurobiol..

[174] Nakase T., Naus C.C. (2004). Gap junctions and neurological disorders of the central nervous system. Biochim. Biophys. Acta.

[175] Kielian T. (2008). Glial connexins and gap junctions in CNS inflammation and disease. J. Neurochem..

[176] Dermietzel R., Gao Y., Scemes E., Vieira D., Urban M., Kremer M., Bennett M.V., Spray D.C. (2000). Connexin43 null mice reveal that astrocytes express multiple connexins. Brain Res. Brain Res. Rev..

[177] Norden D.M., Trojanowski P.J., Villanueva E., Navarro E., Godbout J.P. (2016). Sequential activation of microglia and astrocyte cytokine expression precedes increased Iba-1 or GFAP immunoreactivity following systemic immune challenge. Glia.

[178] Martin H.L., Santoro M., Mustafa S., Riedel G., Forrester J.V., Teismann P. (2016). Evidence for a role of adaptive immune response in the disease pathogenesis of the MPTP mouse model of Parkinson’s disease. Glia.

